# Effects of Phosphatidylserine Source of Docosahexaenoic Acid on Cerebellar Development in Preterm Pigs

**DOI:** 10.3390/brainsci10080475

**Published:** 2020-07-23

**Authors:** Daniel Chizhikov, Randal K. Buddington, Igor Y. Iskusnykh

**Affiliations:** 1 School of Health Studies, University of Memphis, Memphis, TN 38152, USA; danielchizhikov@gmail.com (D.C.); rkb.btf@gmail.com (R.K.B.); 2 White Station High School, Memphis, TN 38152, USA; 3 Babies Taking Flight, Memphis, TN 38117, USA; 4 Department of Anatomy and Neurobiology, University of Tennessee Health Science Center, Memphis, TN 38163, USA

**Keywords:** preterm infants, brain development, phosphatidylserine, docosahexaenoic acid (DHA), cerebellum, pig, granule cells, proliferation, apoptosis

## Abstract

Preterm birth, a major contributor to infant mortality and morbidity, impairs development of the cerebellum, the brain region involved in cognitive processing and motor function. Previously, we showed that at term-equivalent age, preterm pigs that received formula supplemented with docosahexaenoic acid (DHA) esterified to phosphatidylserine (PS) had cerebellar weights similar to those of newborn term pigs and were heavier than control preterm pigs. However, whether PS-DHA promotes the development of specific cerebellar cell populations or enhances key developmental processes remains unknown. Here we investigated the effects of the PS-DHA on development of the cerebellum in preterm pigs delivered via caesarean section and reared for ten days on a milk replacer with either PS-DHA (experimental group) or sunflower oil (control group). Upon necropsy, key cerebellar populations were analyzed using immunohistochemistry. Consumption of PS-DHA was associated with the expansion of undifferentiated granule cell precursors and increased proliferation in the external granule cell layer (EGL). Preterm pigs that received PS-DHA also had significantly fewer apoptotic cells in the internal granule cell layer (IGL) that contains differentiated granule neurons. PS-DHA did not affect the number of differentiating granule cells in the inner EGL, thickness of the inner EGL, density of Purkinje cells, or Bergmann glial fibers, or diameter of Purkinje cells. Thus, PS-DHA may support cerebellar development in preterm subjects by enhancing proliferation of granule cells, a process specifically inhibited by preterm birth, and increasing the survival of granule cells in the IGL. These findings suggest that PS-DHA is a promising candidate for clinical studies directed at enhancing brain development.

## 1. Introduction

Preterm birth affects ~10% of newborns worldwide [1]. It is the primary reason for infant mortality and a significant risk factor for long-term negative neurodevelopmental outcomes, such as impaired motor skills and cognitive function [1,2]. Studies of human patients and animal models reveal that preterm birth impedes growth of the cerebellum, the brain region that regulates motor-coordination and is involved in cognitive processing. Hence, some neurological deficits of preterm infants arise because of disruption of the cerebellar developmental program [3,4,5]. 

Human cerebellar neurogenesis occurs over a prolonged period, beginning in the first trimester of pregnancy and continuing after birth [6,7,8]. The fetal cerebellum arises in the anterior hindbrain and harbors two germinal zones that contain proliferating progenitors: the rhombic lip, which generates glutamatergic granule neurons (granule cells) and the ventricular zone, which generates Purkinje neurons and the molecular layer interneurons and glia [6,9,10]. During embryonic development, proliferating granule precursors exit the rhombic lip and migrate towards the cerebellar pial surface, forming the external granule cell layer (EGL) [11,12]. Granule precursors amplify in the outer part of the EGL. Then, after exiting the cell cycle and beginning differentiation, they translocate to the inner EGL. Finally, differentiating granule neurons migrate through the molecular layer lengthways along fibers of Bergmann glia towards the internal granule cell layer (IGL), the domain harboring differentiated granule neurons [6]. In humans and large animals, the cerebellar growth spurt, which involves extensive proliferation and migration of granule cells, begins in the third trimester of pregnancy. Because of this, the cerebellum is particularly susceptible to adverse factors resulting from preterm birth [13,14]. Indeed, human and animal studies reveal that prematurity and early exposure to the extrauterine environment compromises several major cerebellar cell populations, including granule cells, Purkinje cells, and Bergmann glia [3,4,15]. The potential lifelong impacts emphasize the need for therapeutic strategies that alleviate the compromised cerebellar development associated with preterm birth. 

The developing brain is sensitive to nutritional support [16]. Thus, optimizing early nutrition is a promising strategy for improving cerebellar development of infants born preterm. One promising group of supplements for enhancing neurodevelopment is polyunsaturated fatty acids, including docosahexaenoic acid (DHA). DHA is a structural component of cell membranes in the brain and is predominantly accumulated in the fetal body during the third trimester of pregnancy [17,18]. Because of early birth, preterm infants are frequently deficient in DHA [19]. Several studies have reported an association between the concentration of DHA in preterm infants or DHA supplementation to preterm infants with higher short-term cognitive performance. However, the long-term outcomes remain inconclusive, possibly because of inconsistencies in doses, formula composition, postnatal environment, and other confounding factors [19,20,21,22].

Notably, throughout lactation, human milk contains DHA esterified to phosphatidylserine (PS), a lipid that is critical for the growth and communication of neurons [23]. The amount of DHA that accumulates in the brain is higher when DHA is conjugated to PS, compared to triacylglycerol, likely because of the superior ability of PS-DHA to cross the blood–brain barrier [24,25]. Recently, using preterm pigs as a translational model for preterm infants, we showed that the use of a formula supplemented with PS-DHA results in cerebella that are heavier than those of control preterm pigs and not significantly smaller than those of pigs born at term [16]. This suggests that PS-DHA helps to promote cerebellar growth after preterm birth, but whether it enhances the development of specific cerebellar populations or key cerebellar developmental processes remains unknown. Of particular interest is the understanding if PS-DHA normalizes the development of granule cells and Bergmann glia that we discovered are reduced in preterm pigs [4].

In this paper, by analyzing preterm pigs under highly controlled experimental conditions, we show that PS-DHA does not affect the size and density of cerebellar Purkinje cells or Bergmann glia, but does promote granule cell proliferation and reduces apoptosis in the IGL.

## 2. Materials and Methods

### 2.1. Experimental Design, Preterm Pig Model, and Tissue Collection

Live animal work was performed according to a protocol approved by the Institutional Animal Care and Use Committee of the University of Memphis (#748, approved: 15 June 2015). All the pigs analyzed in the current study were obtained from pathogen-free artificially inseminated sows of a consistent genetic lineage. Preterm pigs were obtained via caesarean section at gestation day 105 (which corresponds to 91% of 115-day term). This stage of development is relevant to preterm infants born at week 32 [16]. A feeding tube and an umbilical artery catheter (Umbili-Cath, Utah Medical Products, Midvale, UT, USA) were placed in each preterm pig. First, all pigs received 5 mL of sow plasma via the umbilical catheter to provide maternal antibodies, compensate for an absence of colostrum and to transfer passive immunity. Then, each pig received a parenteral nutrition solution via an umbilical catheter for 16–18 h until the morning after delivery, when pigs were randomly assigned to experimental and control groups. After being assigned to the experimental or control group, pigs began receiving an identical commercially available liquid milk replacer (Soweena Litter Life, Middleton, WI, USA) via a feeding tube every 3 h (20 mL per kg of body weight). The milk replacer fed to the experimental group was supplemented with PS-DHA (InCog; Enzymotec Ltd., Migdal HaEmek, Israel) at a concentration of 190 mg per 100 mL. The milk replacer fed to the control group was supplemented with the same amount of sunflower oil. Both control and experimental pigs were reared for ten days, when they were euthanized, and the brain was removed from the skull. The cerebellum was dissected and cut into sagittal slices of approximately 1 cm thickness for immunohistochemistry. 

### 2.2. Immunohistochemistry

The slices of cerebella were fixed in 4% paraformaldehyde at 4 °C for 48 h. After that, tissue samples were washed three times (1.5 h each) in phosphate-buffered saline (PBS) at 4 °C and sunk in 30% sucrose in 1x PBS for 14 h at 4 °C. The cerebellar slices were placed in cryomolds, embedded in optimal cutting temperature (OCT) medium (Sakura, Torrance, CA, USA) on dry ice, and sectioned sagittally at 12 µm using a Leica Cryostat. Prior to performing immunohistochemical staining, the slides were dried for 25 min, washed in 1x PBS (3 times, 10 min each), and placed in a blocking solution containing 1% goat serum and 0.1% Triton X100. Primary antibodies were applied for 16 h at 4 °C; then, slides were washed in PBS and incubated with appropriate species-specific secondary antibodies for 1.5 h at 22 °C. We used the following primary antibodies: mouse anti-Tag1 (Developmental Studies Hybridoma Bank, University of Iowa), mouse anti-NeuN (clone A60, Sigma Aldrich, catalog # MAB377), rat anti-phospho S28 histone H3 (Abcam, catalog # ab90543), rabbit anti-cleaved Caspase-3 (Cell Signaling, catalog # 9664T), rabbit anti-Calbindin D-28k (Swant, catalog #CB-38), mouse anti-Ki67 clone B56 (BD Pharmingem, catalog # 556003), and rabbit anti-GFAP (Dako, catalog #Z0334). Species-specific secondary Alexa 488- or Alexa 594-conjugated antibodies were purchased from Invitrogen (Carlsbad, CA, USA). 

### 2.3. Imaging, Cell Counts, and Statistical Analysis

Images were captured with a Zeiss fluorescent microscope. For consistency, all cell quantifications and measurements were performed using sections from medial cerebellar vermis. In each sample, cells were counted in 5 sections separated by at least 60 μm. In each section, cells or fibers were counted in segments of the specified below length, which were randomly selected at the tip of lobe V (anterior cerebellum) and lobe VIII (posterior cerebellum) (one segment per lobe per section). Then an average was calculated, first per section and then per particular sample. The numbers of NeuN+ and Ki67+ cells were determined in 100-μm-long segments of the EGL. Quantification of (Calbindin+) Purkinje cells was performed per 500-µm-long segments of the Purkinje cell layer as reported [4]. The thickness of the Tag1 layer and the diameter of Purkinje cells (in 50 Purkinje cells randomly selected in each cerebellum) were measured with ImageJ software (NIH, USA). Bergmann glial fibers were identified based on GFAP immunohistochemistry and quantified in 200-μm-long segments of the molecular layer. The number of mitotic (phosphorylated histone H3, pH3+) cells was calculated per 1-mm-long segments of the EGL as described [4]. Apoptotic (active Casp3+) cells were quantified per mm^2^ of the IGL. For consistency, all cell counts and measurements were performed by the same investigator, D.C., who was blinded to the group allocation (experimental or control) of the samples. Differences between the experimental and control groups of preterm pigs were evaluated with a two tailed *t*-test (GraphPad Prism8 software, GraphPad Software Inc, San Diego CA, USA). Results are reported as means ± standard deviation (sd); *p* values < 0.05 were considered statistically significant.

## 3. Results

Since PS-DHA increases cerebellar weight in preterm pigs [16], we aimed to investigate the mechanism by which PS-DHA enhances cerebellar development by comparing preterm pigs fed for 10 days with a milk replacer with either PS-DHA (experimental group) or sunflower oil (control group). At the time of necropsy, all pigs had a normal physical appearance without any obvious differences for behavior or motor activity. Macroscopic and microscopic examinations did not reveal any signs of brain injury or hemorrhage.

### 3.1. PS-DHA Promotes Proliferation of Cerebellar Granule Cell Precursors in Pigs Born Preterm

We began our analysis with granule cells because they are critical for proper cerebellar functioning and are reduced at term-equivalent age in pigs born preterm relative to newborn term pigs [4]. We analyzed the numbers of both undifferentiated and differentiating granule cells in the EGL, the location where granule cells are generated. Ki67 immunohistochemistry was performed to identify undifferentiated granule cell precursors populating the outer EGL [4,26]. NeuN immunohistochemistry was used to identify differentiating granule cells that populate the inner EGL. Finally, Tag1 immunohistochemistry was used to evaluate the thickness of the inner EGL [4,27,28]. 

**Figure 1:**
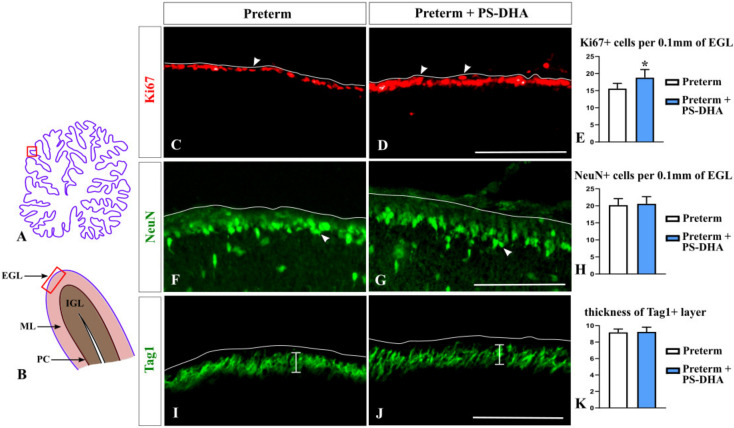
Expansion of undifferentiated (Ki67+) cerebellar granule cell precursors in preterm pigs fed by PS-DHA. (**A**,**B**) Schematic of midsagittal section of the newborn pig cerebellum (**A**). Panel B shows a higher magnification of the region boxed in panel A. EGL, external granule cell layer, ML, molecular layer, PC, Purkinje cell layer, IGL, internal granule cell layer. Panels C, D, F, G, I, J display immunostained sections that correspond to the region shown by the box in panel B. The outer cerebellar surface is depicted by the white line. Sections shown in panels C, D, F, and G were photographed under 10× magnification. Sections shown in panels I and J were photographed under 20× magnification. (**C**–**E**) Undifferentiated progenitors in the EGL identified by Ki67 immunostaining (arrowheads in (**C**,**D**)) were increased in the number in preterm pigs that received PS-DHA (**E**). *n* = 5 pigs per group, * *p* < 0.05, (**F**–**H**) Arrowheads point to differentiating (NeuN+) progenitors in the EGL (**F**,**G**). The numbers of NeuN+ EGL cells were similar in the control and experimental groups (**H**). *n* = 5 pigs per group. (**I**–**K**) Vertical bars show the thickness of the Tag1+ layer in the EGL that contains pre-migratory granule cells (**I**,**J**). The thickness of the Tag1+ layer was not different between the control and experimental groups (**K**). *n* = 5 pigs per group. Scale bars: 100 μm (**C**,**D**,**F**,**G**), 50 μm (**I**,**J**).

Cell counts revealed an increased population of Ki67+ cerebellar granule precursors in preterm pigs that received PS-DHA relative to preterm controls (18.8 ± 2.38 cells per 100 µm of the EGL in PS-DHA preterm pigs versus 15.6 ± 1.51 cells in control preterm pigs, *p* < 0.05) (Figure 1A–E). 

In contrast, PS-DHA fed and control preterm pigs had comparable numbers of NeuN+ differentiating neurons in the EGL (20.6 ± 2.07 cells per 100 µm of the EGL in PS-DHA fed preterm pigs versus 20.2 ± 1.92 cells in control preterm pigs, *p* > 0.05) (Figure 1F–H). Likewise, the thickness of the inner Tag1+ EGL was similar between the PS-DHA fed group and control preterm pigs (9.24 ± 0.57 per 100 µm of the EGL in PS-DHA fed preterm pigs versus 9.18 ± 0.4 in control preterm pigs, *p* > 0.05) (Figure 1I–K).

**Figure 2:**
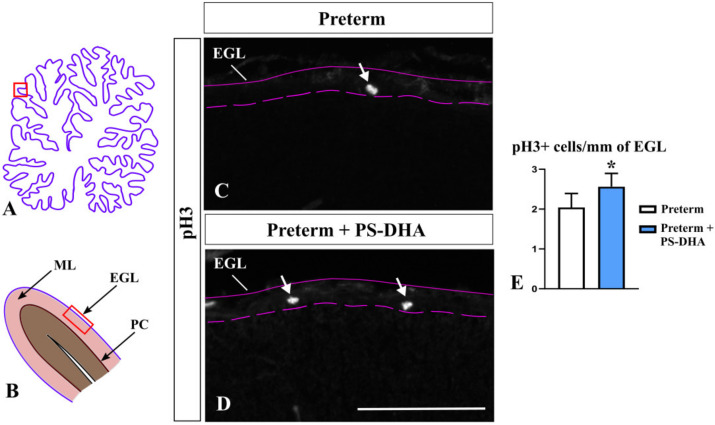
PS-DHA increases mitotic (pH3+) granule precursors in the preterm pig cerebellum. (**A**,**B**) Schematic of midsagittal section of the newborn pig cerebellum (**A**). Panel B shows a higher magnification of the region boxed in panel A. EGL, external granule cell layer, ML, molecular layer, PC, Purkinje cell layer. Panels C and D display sections immunostained against pH3, photographed under 10× magnification, which correspond to the region shown by the box in panel B. (**C**–**E**) Arrows point to mitotic (pH3+) granule precursors in the EGL (**C**,**D**), which were increased in preterm pigs that received PS-DHA relative to control group of preterm pigs (**E**). *n* = 5 pigs per group, * *p* < 0.05. Pink solid and dashed lines show the outer and inner boundaries of the EGL, respectively. Scale bar: 100 μm.

The number of granule cells in the cerebellum is tightly controlled by multiple molecular mechanisms, many of which regulate proliferation [29]. We assessed the proliferation of granule cell precursors using immunohistochemistry against phosphor histone H3 (pH3), which specifically labels mitotic cells [30]. Interestingly, pH3+ cells were more numerous in the EGL of PS-DHA pigs compared to control preterm pigs (2.56 ± 0.3 cells per 1 mm of the EGL in PS-DHA fed preterm pigs versus 2.04 ± 0.4 cells in control preterm pigs, *p* < 0.05) (Figure 2) showing that PS-DHA supplementation promotes proliferation in the EGL. Thus, while PS-DHA does not affect the number of differentiating granule cells in the EGL and the thickness of the internal EGL, it promotes the expansion of granule cell precursors in the preterm cerebellum. 

### 3.2. PS-DHA Promotes Cell Survival in the IGL of Pigs Born Preterm

**Figure 3:**
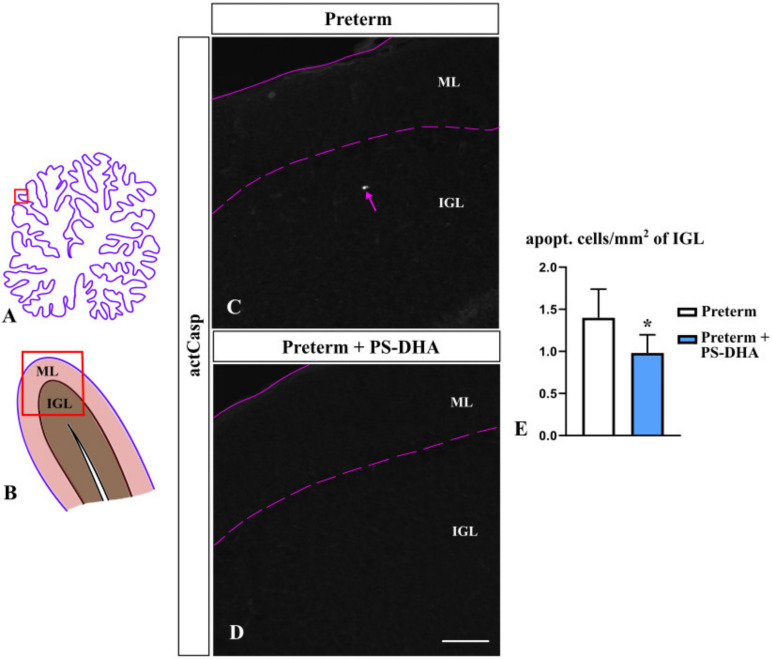
Decreased apoptosis in the IGL (internal granule cell layer) of preterm pigs that received PS-DHA. (**A**,**B**) Schematic of midsagittal section of the newborn pig cerebellum (**A**). Panel B shows a higher magnification of the region boxed in panel A. ML, molecular layer, IGL, internal granule cell layer. Panels C and D display sections immunostained against activated Caspase 3, photographed under 10× magnification, which correspond to the region shown by the box in panel B. (**C**–**E**) The number of apoptotic cells (act Casp+ cells, pointed by pink arrow in panel C) was lower in the IGL of preterm pigs that received PS-DHA relative to control preterm pigs (**E**). *n* = 5 pigs per group, * *p* < 0.05. Pink solid and dashed lines show the outer cerebellar surface and the molecular layer (ML)/IGL boundary, respectively. Scale bar: 100 μm.

Appropriate cerebellar growth depends not only on the proliferation of granule cells, the most numerous neurons in the cerebellum, but also on their rate of apoptosis [4]. To investigate whether PS-DHA affects apoptosis, we measured the number of cells immunopositive for activated caspase 3 in the IGL, the cerebellar layer that contains mature granule cells [10,31]. We found significantly fewer apoptotic cells in the IGL of PS-DHA fed preterm pigs relative to control preterm pigs (0.98 ± 0.22 cells per mm^2^ of the IGL in PS-DHA fed preterm pigs versus 1.4 ± 0.34 cells in control preterm pigs, *p* < 0.05) (Figure 3). Thus, PS-DHA protects cells against apoptosis in the IGL of preterm pigs.

### 3.3. Purkinje Cells Are Not Affected by PS-DHA Supplementation of Preterm Pigs

**Figure 4:**
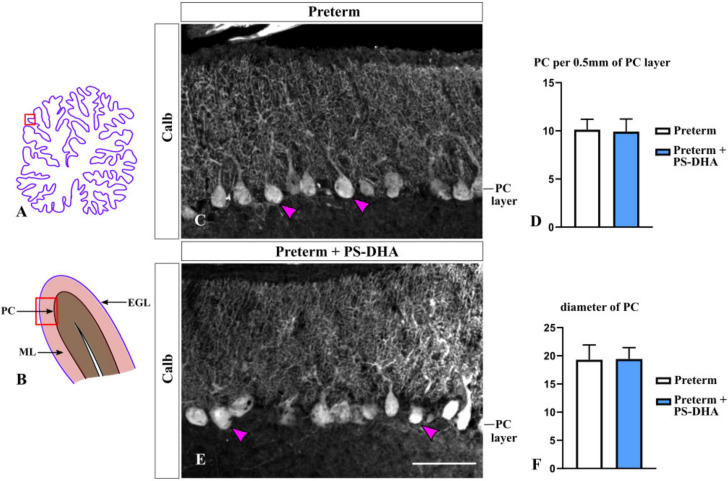
PS-DHA does not affect the density or size of Purkinje cells in the preterm pig cerebellum. (**A**,**B**) Schematic of midsagittal section of the newborn pig cerebellum (**A**). Panel B shows a higher magnification of the region boxed in panel A. EGL, external granule cell layer, ML, molecular layer, PC, Purkinje cell layer. Panels C and E display sections immunostained against Calbindin, photographed under 10× magnification, which correspond to the region shown by the box in panel B. (**C**,**E**) Arrowheads point to Purkinje cells. (**D**,**F**) Quantification analysis revealed a similar density and diameter of Purkinje neurons in preterm pigs that received PS-DHA and the control group of preterm pigs. *n* = 5 pigs per group. Scale bar: 100 μm.

In the cerebellum, Purkinje cells are the only output from the cerebellar cortex and non-autonomously promote proliferation of granule cell precursors [32,33]. Thus, we evaluated Purkinje cells by performing immunohistochemistry against their specific marker Calbindin [34] in preterm pigs that received PS-DHA relative to those that received a control milk replacer. The density of Purkinje cells was similar between the PS-DHA and control groups of preterm pigs (9.92 ± 1.31 per 500 µm of the Purkinje cell layer in PS-DHA fed preterm pigs versus 10.12 ± 1.08 cells in control preterm pigs, *p* > 0.05) (Figure 4A–D). Furthermore, there was no difference in the diameter of Purkinje cells between the PS-DHA and control groups of preterm pigs (19.43 ± 2.01 µm in PS-DHA fed preterm pigs versus 19.3 ± 2.61 µm in control preterm pigs, *p* > 0.05) (Figure 4C,E,F).

### 3.4. Bergmann Glia Are Not Affected by PS-DHA Supplementation of Preterm Pigs

**Figure 5:**
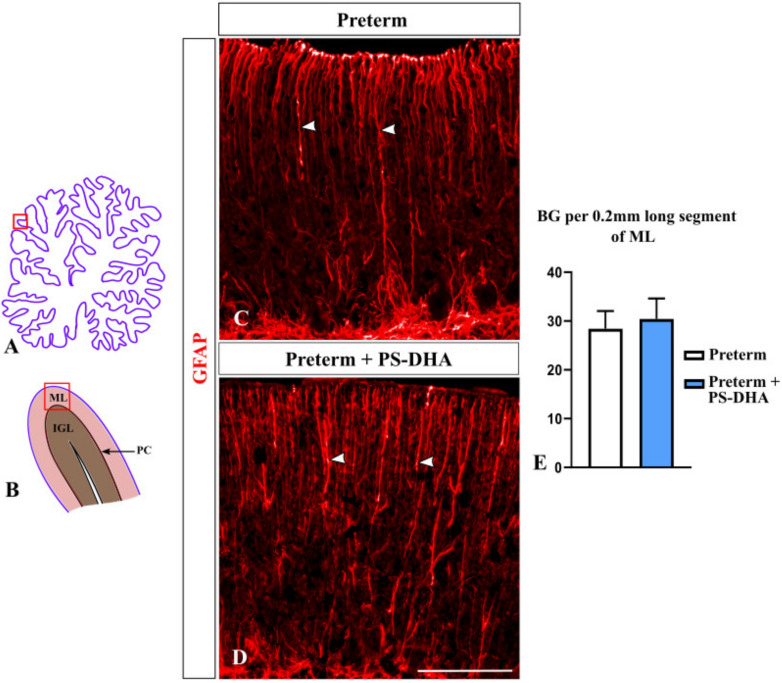
PS-DHA does not affect Bergmann glial fibers in the preterm pig cerebellum. (**A**,**B**) Schematic of midsagittal section of the newborn pig cerebellum (**A**). Panel B shows a higher magnification of the region boxed in panel A. ML, molecular layer, PC, Purkinje cell layer, IGL, internal granule cell layer. Panels C and E display sections immunostained against GFAP, photographed under 10× magnification, which correspond to the region shown by the box in panel B. (**C**,**D**) Arrowheads point to GFAP+ Bergmann glial fibers. (**E**) Quantification analysis did not reveal a significant difference in the number or Bergmann glial fibers between preterm pigs that received PS-DHA compared to the control group of preterm pigs. *n* = 5 pigs per group. Scale bar: 100 μm.

During cerebellar development, Bergmann glial fibers serve as guides during granule cell migration from the EGL to the IGL [35]. Preterm birth decreases the density of Bergmann glial fibers in both humans and animal models [3,4]. Thus, we investigated whether PS-DHA affects Bergmann glia in our preterm pigs using anti-GFAP immunohistochemistry [36]. The density of Bergmann glial fibers was similar in preterm pigs that received PS-DHA relative to control preterm pigs (30.4 ± 4.21 fibers per 200 µm in PS-DHA preterm pigs versus 28.4 ± 3.64 fibers in control preterm pigs, *p* > 0.05) (Figure 5). Thus, PS-DHA did not affect Bergmann glia in the cerebellum of preterm pigs.

## 4. Discussion

Currently, more than 90% of preterm newborns survive the neonatal period, but many exhibit long-lasting negative neurological outcomes such as sensory, motor, and cognitive deficits [37,38,39,40]. Recent findings demonstrate that some neurological deficits appear because of impaired cerebellar development, resulting in a cerebellum reduced in size in both human patients and animals delivered preterm [4,16,41,42,43]. Since brain development in general and cerebellar development in particular are sensitive to nutrition, in the current study we tested the effect of PS-DHA on cerebellar development using the pig as a translational animal model. Our data indicate that feeding preterm pigs with a milk replacer supplemented with PS-DHA enhances the proliferation of cerebellar granule cell precursors, the process inhibited by preterm birth [3,4], and improves survival of cells in the IGL, without any negative effects on the morphology or abundance of other cerebellar cells, including Purkinje neurons and Bergmann glia. 

The reduced cerebellar growth after preterm birth is considered to be mediated largely by a decreased proliferation of granule cell precursors, which results in fewer mature granule cells [4]. Appropriate numbers of granule cells are critical for proper cerebellar function. Thus, increasing the proliferation of granule cell precursors by supplementing pigs born preterm with PS-DHA is likely to be beneficial for cerebellar growth and function. Both PS and DHA are important components of neuronal membranes [44,45,46]. Accumulation of DHA in the fetal brain happens largely during the third trimester of pregnancy, and its concentration is frequently reduced in preterm infants [19]. The endogenous concentration of DHA and DHA supplementation via milk positively correlate with the short-term cognitive performance of preterm infants [19,20,21,22]. The cerebellar granule cells establish firing properties of Purkinje cells, which provide the only output from the cerebellar cortex [47]. The cerebellum is involved in cognition [48], and two-photon calcium imaging revealed that granule cells convey information about the expectation of reward [49]. Thus, it is possible that the higher cognitive performance of preterm infants who received or naturally had higher concentrations of DHA is mediated by proper development (proliferation) of cerebellar granule cells. 

The proliferation of cerebellar granule cell precursors is regulated by multiple pathways. Two major types of molecules that promote the proliferation of granule precursors are Shh secreted from Purkinje cells and Jag1 [32,50,51]. Reduced production of Shh has been described in the postmortem cerebellar samples of preterm infants, while a reduced Jag1 expression has been found in the cerebellum of preterm pigs [3,4]. Future analysis is required to determine whether PS-DHA enhances proliferation of granule precursors by acting via the aforementioned or other signaling pathways.

In addition to the proliferation rate of progenitors, differentiation and survival are critical determinants of the number of granule neurons in the cerebellum. We did not see a notable difference in the number of NeuN+ granule cells in the EGL or in the thickness of the inner Tag1+ EGL between the PS-DHA and control preterm pigs, suggesting that PSA-DHA does not affect differentiation of granule cells. In contrast, we observed fewer apoptotic cells in the IGL of preterm pigs that received PS-DHA, suggesting that it promotes survival of granule neurons. Interestingly, in utero, fetuses develop in hypoxic conditions. Precocious exposure to the ex-utero environment leads to oxidative stress, which is known to trigger apoptosis and compromise cerebellar development [52]. Since DHA is a known antioxidant [53], it is possible that it decreases apoptosis of neurons in the IGL by reducing oxidative stress. 

Although preterm birth and early exposure to the extrauterine environment have been reported to compromise the development or survival of both Purkinje cells and Bergmann glia, we did not find any differences between preterm pigs that received PS-DHA and the control preterm pigs in density or diameter of Purkinje cells, or density of Bergmann glial fibers. Thus, these two cerebellar populations in the preterm cerebellum are unlikely to benefit from the supplementation of PS or DHA. 

Importantly, all the cerebellar phenotypes that we describe herein were analyzed at the term-equivalent stage, following 10 days of PS-DHA supplementation. Although the PS-DHA used in the current study led to a notable increase in the proliferation of granule cell precursors and decreased apoptosis in the IGL, future analysis is required to determine whether these changes are translated into an increased number of mature granule neurons in the adult cerebellum and/or enhanced functional performance. Further, in this study we analyzed only medial cerebellar vermis rather than performing unbiased stereological cell counts in the entire cerebellum. Additional analysis is required to determine whether the phenotypes that we describe are unique to the medial vermis or involve cerebellar hemispheres as well. 

## 5. Conclusions

This study shows, for the first time, that PSA-DHA, at least transiently, enhances cell proliferation in the EGL and improves survival of cells in the IGL in the preterm cerebellum, supporting the development of granule cells, the population affected by preterm birth.
